# Prevention of Cardiomyopathy in Transfusion-Dependent Homozygous Thalassaemia Today and the Role of Cardiac Magnetic Resonance Imaging

**DOI:** 10.1155/2009/964897

**Published:** 2009-04-29

**Authors:** Athanassios Aessopos, Vasilios Berdoukas, Maria Tsironi

**Affiliations:** First Department of Medicine, University of Athens, “Laiko” Hospital, Goudi, Athens 11527, Greece

## Abstract

Transfusion and iron chelation therapy revolutionised survival and reduced morbidity in patients with transfusion-dependent beta thalassaemia major. Despite these improvements, cardiac disease remained the most common cause of death in those patients. Recently the ability to determine the degree of cardiac iron overload, through cardiac magnetic resonance imaging (CMR) has allowed more logical approaches to iron removal, particularly from the heart. The availability of two oral chelators, deferiprone and deferasirox has reduced the need for the injectable chelator deferrioxamine and an additional benefit has been that deferiprone has been shown to be more cardioprotective than deferrioxamine. This review on the prevention of cardiac disease makes recommendations on the chelation regime that would be desirable for patients according to their cardiac iron status as determined by CMR determined by CMR. It also discusses approaches to chelation management should CMR not be available.

## 1. Introduction

In beta thalassaemia major, transfusion and iron chelation therapy have significantly improved survival and reduced morbidity [1, 2]. However, heart complications still represent significant morbidity and remain the leading cause of mortality in transfusion-dependent thalassaemia (TM) patients [2]. In some cases this was because of the difficulty in accepting the chelation treatment, which was cumbersome [3], and also occurred even in some patients who accepted the chelation therapy well [4, 5].

Today, three chelators are available for body iron reduction in transfusion-dependent anaemias. Deferrioxamine, the injectable form, has been available for almost 40 years, deferiprone, the first oral iron chelator, was licensed in Europe since 1999 and recently deferasirox has been licensed in many countries.

Knowledge derived by recent MRI (CMR) studies which also assessed cardiac function showed that all patients with reduced LV function had cardiac iron overload and in many cases this was severe [6–9]. Therefore, the ability to assess cardiac iron by MRI and greater choices in iron chelation have revolutionised the approach to management of transfusion iron overload with the ability to appropriately tailor iron chelation therapy and promises improved survival.

The suggested iron chelation regimes available till recently, that is, with desferrioxamine at 30–40 mg/kg body weight per infusion, 8–10 hours per infusion 5–7 days per week, improved survival and reduced morbidity [10]. However, varying compliance and other factors resulted in continuing presentation of cardiac dysfunction and premature cardiac deaths.

## 2. Iron Load and Predictive Factors of Heart Injury from Iron

For many years, prediction of potential heart iron injury in TM patient was considered necessary in order to assess the efficacy of the treatment regimes, particularly the chelation therapy and to propose any modification. Ferritin levels, and liver iron concentrations (LICs) were the standard surrogate markers used. Assessment of cardiac function particularly by echocardiography is certainly important but of limited value, because, usually, once changes are seen, the patients already have established cardiomyopathy though, in some circumstances, there are some parameters that can be determined with echocardiography that can predict that the patient is iron overloaded such as the total diameter index. The latter is calculated by adding the ASE end-diastolic measurements of the RV diameter, the intraventricular septum thickness, the LV diameter, and the posterior wall thickness divided by BSA. A total diameter index (Tdi) >5.57 cms/m^2^ is highly specific for predicting cardiac iron (91.4%) but only had a low sensitivity (31.8%) [11].

In two recent studies, one of which assessed most recent [9] and one of which assessed highest, lowest, mean 5 year, and most recent ferritin [8], there was a statistically significant relationship of ferritin to MRI-assessed cardiac iron but no predictive value between the two indices.

Until recently, the LIC was given great significance with respect to the risk of cardiac disease and it was recommended that levels >12 mg/gm dry weight were associated with cardiac death [12]. However, in one study on 58 transfusion-dependent patients, aged between 10–45 years, the majority of whom had thalassaemia major and who were on regular chelation therapy with desferrioxamine, it was demonstrated that LIC (by biopsy) was not related to cardiac dysfunction as assessed by stress MUGA, [13] in that both the resting LVEF and the increase in LVEF after exercise stress showed no statistical relationship to the LIC. In another large study from Torino, 652 patients with thalassaemia major aged between 1–27 years, the LIC, as estimated by SQUID and echocardiography findings, showed similar lack of relationship [14]. These findings have been elucidated by recent MRI studies. One did not show statistical significance between LIC and cardiac iron and the others did, while there was no predictive value between them [6–9]. Therefore, using LIC as a predictor of cardiac mortality can be misleading. Caution should be exercised, particularly in patients with satisfactory ferritin levels and LIC as if they are low, both the patients and their physician may believe that the patient is protected from heart disease and it is not realised that patients with such levels may have excessive cardiac iron and need intensification of chelation therapy [8]. Irrespective of this, major efforts should be made to maintain low LICs because high levels are potentially dangerous and are associated with other morbidities such as increased risk of siderophore bacterial infections, cirrhosis, and hepatoma.

As there was no particular examination giving a real indication of cardiac risk, the ability to determine cardiac iron, was therefore, crucial. Cardiac biopsy is invasive and inaccurate [15, 16], therefore, the ability to assess cardiac iron noninvasively, reproducibly, and accurately was imperative. Cardiac magnetic resonance imaging (CMR) has offered that capability. A number of studies have demonstrated the value of CMR in indirect assessment of cardiac iron overload (T2*) and function parameters [17–21]. Many other centres are instituting either the same or similar MRI techniques. The results appear to be comparable using different machines and in different countries [22]. They are reproducible and robust, provided that the T2* method is used and the area measured is the intraventricular septum [23]. The classification of patients is that those with T2* > 20 msecs are regarded as not having cardiac iron [6, 8, 9]. Those with T2* between 10–20 msec have mild-to-moderate cardiac iron load and those <10 msecs are considered to have heavy cardiac iron load.

## 3. Chelation Treatment for Prevention of Iron Induced Heart Disease

Chelation treatment today should be guided by MRI findings if the technique is available. In the presence of excess cardiac and/or hepatic iron, treatment strategies include increase of the dose and/or frequency of desferrioxamine, switch to oral chelators in maximal permissible doses (deferiprone or deferasirox) depending on the degree of cardiac iron load or to the combination of deferiprone with desferrioxamine, provided there are no contraindications to their use [24]. With respect to hepatic iron removal, the efficacy of the two oral chelators is at least equal to the standard doses of desferrioxamine [25–27]. Recent and ongoing studies have demonstrated that deferiprone, which is a small molecule that permeates all tissues, is more efficient in removing cardiac iron and improving cardiac function than desferrioxamine [25, 26]. Clinical studies with deferasirox are currently ongoing with respect to removal of cardiac iron. As yet, there are no studies with combinations of deferasirox and desferrioxamine so this therapeutic regime cannot be recommended at this stage.

According to the current knowledge and based on the CMR findings, the suggested chelation regimes are as follows.

### 3.1. Acceptable Cardiac Iron

For patients with T2* greater than 20 msec, the therapeutic strategy should be continuation of monotherapy with either desferrioxamine or either of the available oral chelators (deferiprone and desferasirox) with regular followup. For patients convenience, desferrioxamine administration may be converted to either of the two oral chelators.

### 3.2. Mild-to-Moderate Cardiac Iron Loading

T2* values between 10–20 msec are considered to reflect a mild-to-moderately iron-loaded myocardium. Bearing in mind that the patients may be at risk of developing cardiac problems under stress such as infections, clearing myocardial tissue from iron seems to be a rational target. Therefore, combined treatment for these patients should not be a priori excluded. Patients have presented with LV dysfunction at levels of T2* of 15 msec, without any precipitating factors [8]. Therefore, if T2* is ≤15 msec, combination chelation therapy is recommended [24]. However, questions still exist, regarding the frequency and the amount of desferrioxamine administration that is appropriate in a combined regimen. A dose of 35–40 mg/kg/day three-four times weekly combined with deferiprone at a dose of 75 mg/kg/day seems to be reasonable. In patients with T2* 15–20 msec, monotherapy with either deferiprone or deferasirox together with careful followup are available options [25, 26]. As there are no published trials, at present available, on the efficacy of deferasirox in removing cardiac iron, if it is to be prescribed, it is advisable that MRI followup be more frequent than if deferrioxamine and deferiprone are prescribed. Patients treated up to the time of the MRI with desferrioxamine in this category and who availed themselves of that treatment satisfactorily should not be on monotherapy with that compound, as deferrioxamine was inadequate at preventing the iron accumulation in the heart and may indicate some type of resistance to its efficacy in the heart within that patient.

### 3.3. Heavy Cardiac Iron Load 

Patients with T2* < 10 msec are considered to have severe iron overload and this category includes most patients with reduced left ventricular (LV) function. Even those patients with normal ejection fraction in this category are considered to be at a great risk of developing cardiac dysfunction. Thus, all patients in this category have a strong indication for combined chelation treatment. The doses of deferrioxamine should be approximately 50 mg/kg/subcutaneous infusion up to 7 days per week if tolerated and the deferiprone should be given at doses between 75–100 mg/kg/day in three divided doses. If the use of deferiprone is contraindicated, then the patients should be treated with intensive desferrioxamine therapy, either through continuous infusions through an indwelling catheter or by subcutaneous continuous infusions [28].

Any treatment modification should be followed by close monitoring. Should any serious adverse effect present as a consequence of the administration of a particular chelator, appropriate guidelines as to its continued use should be followed.

If treatment has ultimately modified the CMR patient's classification, then it may be adjusted as discussed earlier according to the changes in CMR values.

### 3.4. Guidelines If MRI Is Not Available

In countries where MRI is not available, then all the patients' traditional parameters need to be analysed, (ferritins, liver iron concentrations) as well as ECG and echocardiogram, taking into account the above-discussed limitations. These may serve as a guide to treatment. In general, iron-related cardiomyopathy rarely appears before the age of about 14 years. Therefore, until that age, the choice of chelator that should be recommended depends on those parameters and which of the three chelators will be more acceptable and tolerable for the patient and his/her family.

A recent study from Italy has shown that patients who continued treatment with deferrioxamine had a greater incidence of cardiomyopathy with greater cardiac-related mortality than patients who were changed to deferiprone [29]. This difference should be considered in patients who do not have access to CMR in determining which chelator would be most suitable for patients.

Furthermore, according to the knowledge from MRI studies in countries where followup of patients occurs, up to 65% of patients have cardiac iron load. In Sardinia, 13% had severe cardiac iron overload [9]. In our study, 48% of patients have T2* < 15 msecs [8]. In countries where patients compliance to treatment is inadequate, there was poor availability of chelation and/or the followup was not well organized, the percentage of cardiac iron-loaded patients is likely to be higher. Therefore, for patients who have never had optimal care, it is very likely the patients will have cardiac iron load and intensive chelation as the treatment of choice. In patients who have been poorly chelated, the risk of chelation toxicity is minimal and would only be likely to occur after prolonged therapy, however, it is important to be vigilant for such complications. MRI is more necessary for those patients who have had good chelation therapy but who are at risk of chelation inadequacy with respect to the heart and for those who have had treatment modification in order to follow the efficacy of the changed chelation regime.

## 4. Conclusions on Prevention of Heart Disease

This genetic defect that was formerly almost universally fatal has been revolutionised with the availability of adequate chelation therapy and more recently with other important advances.

It remains important, practically, to aim to maintain low LICs and ferritin levels, particularly as the latter are easily accessible and assessable. Similarly, echocardiography should remain a routine tool as it does have some predictive value and can also be used to monitor patients in whom intensification of chelation therapy has been instituted.

CMR can be particularly helpful in identifying all TM patients at risk of developing heart disease by assessing the cardiac iron load. Chelation therapy can be tailored to remove the excess heart iron. Attention to patient's continuous compliance with adequate chelation is mandatory.

The definite ability to know and reduce cardiac iron as well as improvement in cardiac function that has been reported should certainly lead to even further significant reduction in cardiac mortality and morbidity. In situations in which CMR is not available, data available from other centres can help the clinicians with their decision as to which iron chelator to recommend for each individual patient.
